# Case of Preternatural Labor, Complicated with Convulsions and an Hour-Glass Contraction of the Uterus

**Published:** 1871-03

**Authors:** D. W. Flick

**Affiliations:** West Grove, Iowa


ARTICLE II.—*Case of Preternatural Labor, Complicated with Convulsions and an Hour-Glass Contraction of the Uterus.* By D. W. FLICK, M.D., West Grove, Iowa.

At noon, Dec. 26th, 1870, I was called to see Mrs. W., aged 20; primapara. She is of florid complexion, small frame, 4 ft. 11 in. in height, rather stout built, short neck.

Upon inquiry, I learned she had vomited every day during the whole course of her pregnancy, and that she was also troubled with habitual constipation. Her feet, legs and arms were much swollen, and she had complained of headache, dizziness, and trouble in voiding her urine for two or three weeks.

She had an operation of the bowels in the morning, and had urinated once or twice during the forenoon.

Upon examination per vaginam, I found the os uteri dilated about the size of a half dollar, and situated within an inch and a half or two inches of the ostium vagina, vertex presenting. She was very restless, and vomited once or twice during the afternoon. Pains were migratory, irregular and severe. To lessen their severity, I gave chloroform sparingly.

At 11 o'clock P. M. I found but slight change in the os uteri. I then ruptured a very strong membrane, which was succeeded by an unusually large flow of water. The patient grew very irritable, as the labor was slow and tedious. At 3 o'clock A. M., 27th, passed urine with much difficulty. At 5.20 A. M. she gave birth to a very feeble male child, weighing 6 lbs. 11 ozs., after which she seemed cheerful and in good spirits.

I had suspected another child in utero, and upon further examination found my suspicions confirmed. Vertex presenting. I immediately announced it to one of the ladies present, and in less time than it takes to write it, the patient was seized with convulsions. I then deemed it expedient to rupture the membrane of the second child, which I accordingly did, but pains did not return. I then dispatched a messenger for my forceps, a distance of five miles, and another as soon as possible for Dr. Sawyers, of Unionville. Gave her Brom. Pot. gr. xxv. At 7 o'clock A. M. she had another very hard convulsion, and by the time it had passed off, my forceps had arrived. I immediately chloroformed her, and delivered her of a male child, (not without some difficulty, as a large umbilical cord prevented easy adjustment of the forceps,) weighing 6 lbs. 12 ozs., and very feeble.

I immediately discovered an hour-glass contraction of the uterus, which, however, gave way with little difficulty. Placenta adhered, but easily detached. I think it would weigh 3 lbs. After delivery, the womb contracted tolerably well. There was, however, considerable flooding.

The effects of the chloroform now began to wear off. She spoke to me, and asked if she did not faint twice. I answered in the affirmative. She replied, "I faint very easily," and scarcely had she spoken ere she was seized with another convulsion, which was very severe, and lasted ten minutes. 8 o'clock A. M.

Gave Brom. Pot. gr. x. every ten minutes, and, determining to forestall the next convulsion, used chloroform very freely. But in this I was disappointed, for in a few minutes after 10 o'clock she was seized with another.

In the meantime Dr. Sawyers had arrived, and both rendered me valuable assistance and encouraged me with his timely presence.

We then added Brom. Ammon. gr. xv. to Brom. Pot. gr. xx., and gave every half hour. Kept her under the influence of chloroform, with slight letting up occasionally. Her tongue at this time was much swollen, and somewhat coated. Pulse 90, and very feeble. Temperature natural.

After giving one or two doses of the above prescription, there was so much difficulty in swallowing that we discontinued the Ammonia, and gave Brom. Pot. as before. At 12.50 P. M. she had another convulsion, while completely under the influence of chloroform. At 1.50 another. At 2 o'clock we gave Hydg. Submur. gr. xx, and Morphia gr. ss. At 2.12 she had another convulsion.

Dr. Sawyers then introduced the catheter, and drew off about xij. oz. water.

At 2.32 another convulsion; 2.55 another; 3.30 another; at 5 o'clock another, which proved to be the last.

In accordance with Dr. Sawyers' advice, (his business being such that he could remain no longer with me,) I gave at 8 o'clock P. M., gtt. v. Oleum Tiglii, in a teaspoonfull of castor oil. At this time the urine passed freely.

It was impossible to get her to swallow anything from this time up to 9 o'clock A. M. of the 28th, but she was perfectly comatose for the space of thirteen hours.

At 9 o'clock her bowels moved, urine passed freely, and consciousness partially returned. I commenced Brom. Pot. gr. xx. every hour. Bowels moved at 12 o'clock, and a large amount of very offensive fœcal matter passed. Patient better. At 2 o'clock P. M. bowels moved again, and at 3.

I had now been absent from home over forty-eight hours, and felt obliged to return, at least for a short time. Patient at this time was very restless, tossing from one side of the bed to the other. Gave gr. iv. Pulv. Opii, and Quinine gr. ij., to be given every six hours. Brom. Pot. every two hours.

Upon my return at 10 o'clock A. M., 29th, found pulse 110, feeble. Extremities cold. Gave Quinine, Opii and Camph., āā gr. ij. every four hours. Applied sinapisms and heated brick. Discontinued Brom. Pot., and gave beef tea and brandy toddy alternately.

Patient became quiet, and slept calmly and naturally. Treatment continued until the morning of the 30th. Pulse was then 90, full and regular. Temperature, 99. She was calm and rational, and could take a little nourishment. Pronounced her out of danger and left her. At last accounts, she and both infants were doing well.

